# Merged testing for colorectal cancer syndromes and re‐evaluation of genetic variants improve diagnostic yield: Results from a nationwide prospective cohort

**DOI:** 10.1002/gcc.23049

**Published:** 2022-05-02

**Authors:** Sara Svensson, Theofanis Zagoras, Christos Aravidis, Marie Stenmark Askmalm, Erik Björck, Åke Borg, Ekaterina Kuchinskaya, Mef Nilbert, Margareta Nordling, Anna Rohlin, Gustav Silander, Kristina Lagerstedt‐Robinson, Samuel Gebre‐Medhin

**Affiliations:** ^1^ Division of Clinical Genetics, Department of Laboratory Medicine Lund University Lund Sweden; ^2^ Department of Clinical Genetics and Pathology Office for Medical Service Lund Sweden; ^3^ Department of Laboratory Medicine Institute of Biomedicine, Sahlgrenska Academy, University of Gothenburg Gothenburg Sweden; ^4^ Department of Clinical Genetics and Genomics Sahlgrenska University Hospital Gothenburg Sweden; ^5^ Department of Clinical Genetics Akademiska University Hospital Uppsala Sweden; ^6^ Department of Molecular Medicine and Surgery Karolinska Institutet Stockholm Sweden; ^7^ Department of Clinical Genetics Karolinska University Laboratory, Karolinska University Hospital Stockholm Sweden; ^8^ Institute of Clinical Sciences, Division of Oncology Lund University Lund Sweden; ^9^ Department of Biomedical and Clinical Sciences, Division of Cell Biology Linköping University Linköping Sweden; ^10^ Department of Radiation Sciences Oncology, Umeå University Umeå Sweden

**Keywords:** colorectal cancer, genetic testing, hereditary, polyposis, syndrome, variant classification

## Abstract

Approximately 5% of patients with colorectal cancer (CRC) have a Mendelian predisposition for the disease. Identification of the disease‐causing genetic variant enables carrier testing and tailored cancer prevention within affected families. To determine the panorama and genetic variation of Mendelian CRC syndromes among referrals at the cancer genetics clinics in Sweden, 850 patients clinically selected for CRC genetic investigation were included in a prospective study that tested for all major hereditary polyposis and nonpolyposis CRC conditions. Genetically defined syndromes were diagnosed in 11% of the patients. Lynch syndrome was predominant (*n =* 73) followed by familial adenomatous polyposis (*n =* 12) and *MUTYH*‐associated polyposis (*n =* 8); the latter of which two patients presented with CRC before polyposis was evident. One patient with a history of adolescent‐onset CRC and polyposis had biallelic disease‐causing variants diagnostic for constitutional mismatch repair deficiency syndrome. Post‐study review of detected variants of unknown clinical significance (*n =* 129) resulted in the reclassification of variants as likely benign (*n =* 59) or as diagnostic for Lynch syndrome (*n =* 2). Our results reveal the panorama of Mendelian CRC syndromes at the cancer genetics clinics in Sweden and show that unified testing for polyposis and nonpolyposis CRC conditions as well as regular reexamination of sequence data improve the diagnostic yield.

## 1 INTRODUCTION

Approximately 5% of patients with colorectal cancer (CRC) have a constitutional disease‐causing genetic variant (DV) that causes autosomal dominant (AD) or autosomal recessive (AR) predisposition to the disease.^1^ Lynch syndrome (LS) is by far the most common known Mendelian CRC condition with a prevalence approximately 1:300, followed by familial adenomatous polyposis (FAP) and *MUTYH*‐associated polyposis (MAP; AR inheritance) with estimated prevalences approximately 1:10 000 to 1:40 000, respectively.^2^, ^3^ There are several less common Mendelian conditions that predispose to CRC, such as juvenile polyposis syndrome (JPS), *PTEN* hamartoma tumor syndrome (PHTS), Peutz–Jeghers syndrome (PJS), polymerase proofreading‐associated polyposis (PPAP), and a handful additional more recently defined conditions.^3^, ^4^ Because DV may have prognostic, therapeutic, and prophylactic implications, screening for DV in selected patients with CRC and/or colorectal polyposis is routine in many health‐care systems. In addition, identification of DV permits carrier testing and personalized health care for relatives. Patients with colorectal polyposis with or without CRC have traditionally been genetically screened FAP and MAP. Conversely, patients with nonpolyposis CRC have traditionally been genetically screened for LS. To date, the outcomes of simultaneous testing for Mendelian CRC have mostly been evaluated in retrospective cohorts of selected or unselected patients.^2^, ^5^, ^6^, ^7^ In this work, we have addressed the panorama and genetic variation of Mendelian CRC syndromes in a prospective national cohort of patients clinically selected for CRC genetic diagnostics.

## 2 MATERIALS AND METHODS

### 2.1 Patients

**FIGURE 1:**
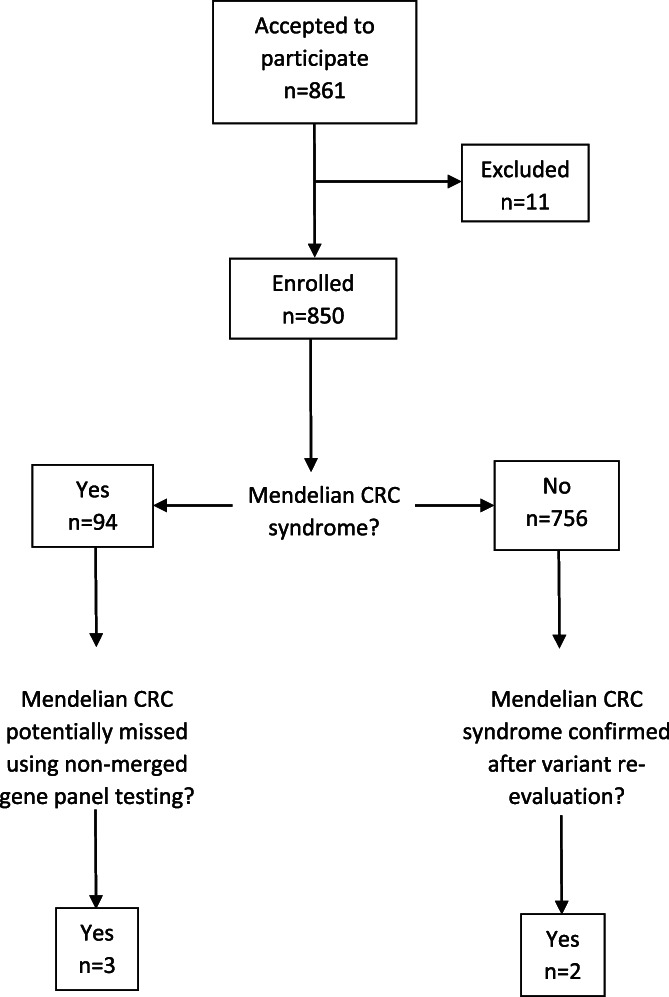
Schematic description of study outline and diagnostic yield

From 2014 to 2019, 861 patients with suspected Mendelian predisposition to CRC were invited to participate in the Swedish‐extended genetic analysis of colorectal neoplasia (SWEN) study, a prospective study with inclusion from all cancer genetics clinics in Sweden, including the university hospitals in Umeå, Uppsala, Stockholm, Linköping, Gothenburg, and Lund (Figure 1). Patients obtained oral and written study information and provided written informed consent. This study was approved by The Regional Ethical Review Board in Lund (application no. 2013/468 and no. 2015/211) and by the Swedish Ethical Review Agency (application no. 2019‐02312). The inclusion criteria of the study were as follows: (1) age of majority (≥18 years), (2) CRC clinical genetic investigation decided upon at a cancer genetics clinic in Sweden according to national clinical guidelines (Supplementary Material 1), and (3) written informed consent. After exclusion of 11 patients due to patient withdrawal (*n =* 5), no sample available (*n =* 2), incomplete molecular genetic analysis (*n =* 1), previously genetically screened for DV in MMR genes (*n =* 2), or inclusion merely on the basis of family history (*n =* 1), 850 patients were successfully enrolled in the study and cataloged regarding gender, neoplasms, colorectal polyps, and age at diagnosis. Records of unspecified numbers of colorectal polyps stating “polyposis”, or “many”, “numerous” or “massive numbers of” polyps were interpreted as 10 or more polyps and denoted polyposis.

### 2.2 Molecular genetic analyses

All patients were screened for DV in genes associated with established Mendelian CRC syndromes including LS (i.e., DNA mismatch repair [MMR] protein‐encoding genes *MLH1*, *MSH2*, *MSH6*, and *PMS2*, and the *MSH2*‐adjecent gene *EPCAM*), FAP (*APC*), MAP, JPS (*BMPR1A* and *SMAD4*), PHTS, and PJS (*STK11*). A majority of the patients (*n =* 702) were also screened for DV in the genes responsible for PPAP (*POLE* gene; *POLD1* gene), and *GALNT12* (putative gene for familial CRC type X; FCCTX), and the *GREM1* gene (upstream duplication only; candidate gene for hereditary‐mixed polyposis syndrome; HMPS). All molecular genetic analyses were performed with genomic DNA extracted from venous blood according to standard laboratory procedures. DNA was sequenced using targeted capture MPS (massive parallel sequencing) assay, either using SureselectXT Custom and sequencing using Illumina HiSeq 2000 (*n =* 616) and analyzed as previously described,^8^ or using AmpliSeq following sequencing using an IonTorrent S5 (*n =* 234) as previously described^9^ and analyzed using IonReporter. All variants identified by capture MPS were confirmed using Sanger sequencing using standard protocols. Samples analyzed with IonTorrent sequencing regarding *PMS2* were also subjected to nested PCR followed by Sanger sequencing as described.^10^ For detection of deletions/duplications, samples analyzed with IonTorrent were also analyzed using multiplex ligation‐dependent probe amplification (MLPA) regarding the genes *APC*, *EPCAM*, *GALNT12*, *MLH1*, *MSH2*, *MSH6*, *MUTYH*, *PMS2*, *PTEN*, *SMAD4*, and *STK11* (MRC‐Holland, Amsterdam, The Netherlands) according to the manufacturer's instructions.

### 2.3 Variant classification

Data were analyzed using Gene Marker (Soft Genetics). Variant classification was performed according to the America College of Medical Genetics (ACMG)^11^ and ClinVar.^12^ Two main categories of variants were reported: DV including variants of class 4 (likely pathogenic variants) and variants of class 5 (pathogenic variants), and variants of unknown significance (VUS; class 3). Likely benign variants (class 2) and benign variants (class 1) were regarded as normal findings and were not reported. DV and VUS were re‐evaluated in October 2021, that is, 2–7 years after initial reporting.

### 2.4 Statistics

For statistical analysis, two‐tailed *t*‐test, chi‐square test of goodness of fit, or chi‐square test of independence were used (https://www.socscistatistics.com). Values of *p* < 0.05 were considered statistically significant.

## 3 RESULTS

### 3.1 Clinical characteristics of cohort

The cohort contained 527 females and 323 males (Table 1). A total of 1025 cancers were recorded with a median age at diagnosis of 52 years (Table 2). Cancer of any type, CRC, and polyposis occurred without significant differences between the sexes (Table 1). More females than males were recorded with ≥2 cancers (Table 1). Females remained supernumerary to males when individuals with ≥2 cancers were excluded (404 females vs. 283 males; *P* = 0.00001). Similarly, females remained supernumerary to males when individuals with breast cancer and reproductive organ cancers were removed (381 females vs. 306 males; *P* = 0.0042). Females were also more numerous than males among patients with solitary CRC and no other diagnosis (292 females vs. 226 males; *P* < 0.040).

**TABLE 1:** Summary of clinical characteristics of all 850 patients by confirmed Mendelian colorectal cancer syndrome

	Total	Sex	Age at diagnosis (years)^a^	Mendelian syndrome			
		Female (%)	Median (range)	LS^b^	FAP^c^	MAP^d^	CMMRD^e^
All patients	850	527 (62)*	49 (16–100)				
Cancer of any type	806	503 (62)	49 (17–100)	73	5	5	1
Colorectal cancer	693	412 (59)	50 (17–100)	61	4	5	1
Polyposis	94	52 (55)	49.5 (16–86)		12	8	1
≥2 Cancers	163	123 (75)**	50 (17–82)	21			
≥2 Colorectal cancers	32	20 (63)	56 (37–81)	8			
Mendelian syndrome	94	51 (54)	45*** (16–81)	73	12	8	1
Syndrome not detected	756	476 (63)	49 (17–100)				

* *P* < 0.00001 for female sex/male sex.

** *P* = 0.001 for ≥2 cancers in females/≥ 2 cancers in males.

*** *P* < 0.01 for Mendelian syndrome/syndrome not detected.

^a^ Age at first cancer or polyposis.

^b^ Lynch syndrome.

^c^ Familial adenomatous polyposis.

^d^ *MUTYH*‐associated polyposis.

^e^ Constitutional mismatch repair deficiency.

**TABLE 2:** Summary of cancers in cohort by confirmed mendelian colorectal cancer syndrome

	Total	Sex	Age (years)	Mendelian syndrome			
		Female (%)	Median (range)	LS^a^	FAP^b^	MAP^c^	CMMRD^d^
All cancers	1025	670 (65)	52 (16–100)	113	5	5	1
Colon	594	353 (59)	51 (17–100)	62	4	4	1
Rectum	138	84 (61)	47 (27–81)	10		1	
Uterus	74	74 (100)	55 (27–82)	19			
Breast	62	61 (98)	53.5 (33–82)	6			
Ovary	37	37 (100)	49 (26–81)	4			
Prostate	16	0 (0)	66 (54–72)	2			
Bladder	12	1 (8)	52 (29–50)	1			
Small bowel	11	6 (54)	50 (32–75)	2			
Stomach	9	2 (22)	48 (36–70)	1			
Kidney	9	5 (56)	65 (38–72)	1			
Pancreas	8	8 (100)	70.5 (55–78)	1			
Ureter	5	1 (20)	55.5 (52–64)	3			
Brain	3	2 (67)	57 (55–67)	1			
Thyroid	2	2 (100)	58 (46–70)		1		
Other	45	34 (76)	48 (17–76)				

^a^ Lynch syndrome.

^b^ Familial adenomatous polyposis.

^c^ MUTYH‐associated polyposis.

^d^ Constitutional mismatch repair deficiency syndrome.

### 3.2 Confirmed Mendelian CRC syndromes

Mendelian CRC syndromes were genetically confirmed in 94 patients (11% of cohort); LS was predominant (*n =* 73), followed by FAP (*n =* 12) and MAP (*n =* 8) (Table 1). One patient with a history of adolescent‐onset CRC and polyposis displayed biallelic DV in an MMR gene diagnostic for constitutional mismatch repair deficiency (CMMRD) (Table 1, patient ID C068; Supplementary Material 2). No patients were genetically confirmed with PPAP, JPS, PHTS, or PJS. All patients with LS had cancer and none of them had polyposis (Table 1). Polyposis was recorded in all patients with FAP, MAP, and CMMRD (Table 1). Age at cancer or polyposis was lower (median age 45 years) in patients with Mendelian CRC syndrome compared to patients with no syndrome detected (median 49 years; Table 1). In two patients with MAP, CRC was diagnosed >10 years before polyposis was discovered (age 51 vs. 64 years; age 39 vs. 50 years, respectively).

### 3.3 Detected genetic variants and variant reclassification

A total of 112 DV and 129 VUS were reported (Table 3). Among the MMR genes, *MSH2* displayed the greatest number of DV diagnostic for LS (Table 3). Two DV in *PMS2* were confirmed as biallelic compound heterozygote variants diagnostic for CMMRD (Table 3, patient ID C068; Supplementary Material 2). In *MUTYH*, 16 DV were biallelic and accordingly diagnostic for MAP, whereas 8 DV were monoallelic representing heterozygote carriers for MAP (Table 3). Post‐study re‐evaluation of all detected variants resulted in the reclassification of 62 variants from VUS to likely benign (*n =* 59), from VUS to DV (*n =* 2), or from DV to VUS (*n =* 1; Table 3; Supplementary Material 2). The most frequent reasons for reclassification from VUS to likely benign were >1/500 allele frequency in any population (*n =* 21) and variants in *POLE* or *POLD1* located outside the exonuclease domain (*n =* 17; Supplementary Material 2).

**TABLE 3:** Summary of detected genetic variants and variant classification by gene

	Total	MLH1	MSH2	MSH6	PMS2	EPCAM	APC	MUTYH	BMPR1A	POLE	POLD1	SMAD4	STK11	GALNT12
All DV^a^	112	20	28	15	11^b^	1	12	24	1^c^					
All VUS^d^	129	11	10	20	13	2	33	6	5	15	7	3	2	2
VUS reclassified as LB^e^	59	4	4	5	8	2	9	6		14	5	2		
VUS reclassified as DV	2	1			1									
DV reclassified as VUS	1								1^c^					

^a^ Disease‐causing variant.

^b^ Including two biallelic variants.

^c^ Present in patient with disease‐causing variant in *EPCAM*.

^d^ Variant of uncertain clinical significance.

^e^ Likely benign variant.

## 4 DISCUSSION

In this work, we have investigated a prospective cohort of patients selected for CRC genetic investigation at the cancer genetics clinics in Sweden during 2014–2019. We aimed to determine the occurrence and genetic variation of Mendelian CRC syndromes using a comprehensive gene panel covering both nonpolyposis and polyposis conditions. A greater number of female patients was observed overall and throughout most clinical subcategories in our cohort, indicating a possible referral or pre‐referral gender bias. Since the literature is scarce on the topic,^13^, ^14^ we can only speculate that women in Sweden for reasons yet to be determined are more prone to request or accept or be offered cancer genetic investigation.

Median age of onset for CRC in our cohort was 49 years, which is >20 years lower than for CRC in the general population in Sweden,^15^ indicating compliance with national referral guidelines for CRC genetic investigation. LS was by far the most frequent Mendelian CRC syndrome detected in our study, followed by FAP and MAP both at approximately 1% levels, that is, mutual ratios for LS, FAP, and MAP repeatedly shown in other studies of selected and unselected patients with CRC and in line with reported prevalences in general populations.^2^, ^3^, ^5^, ^6^, ^7^ Given their rarity, the absence of identified patients with PPAP, PJS, PTHS, and PJS is not surprising as the size of our cohort is limited. Also, as symptoms may present early in life, some patients with these conditions might have been diagnosed before adulthood by pediatricians and thus have escaped our study.

We found one patient with CMMRD, a severe early‐onset multiorgan cancer predisposition syndrome caused by biallelic DV in MMR genes, predominantly in *PMS2*.^16^ Molecular genetic diagnosis of CMMRD can be challenging as it may resemble neurofibromatosis type 1 presenting with café‐au‐lait macules and brain tumors, or FAP or MAP presenting with adenomatous polyposis.^16^ Our case with CMMRD had polyposis and CRC and would have escaped detection if genetic testing had been restricted to CRC polyposis conditions. Indeed, the clinical overlap between Mendelian CRC syndromes is now increasingly recognized,^8^, ^17^ including accumulation of colorectal polyps in some cases of LS^18^ and attenuated forms of MAP and FAP.^19^ None of our patients with LS had ≥10 synchronous polyps. However, two of our patients with MAP presented with early‐onset CRC more than a decade before polyposis was evident. This is in line with the findings of a study done by Nielsen et al., who showed that >30% of patients with CRC and proven biallelic DV in *MUTYH* do not have polyps and approximately 20% have <10 adenomas.^20^ Consequently, as already endorsed by others,^21^ multigene panel testing including all high‐penetrant Mendelian CRC syndromes should be promoted in patients with early‐onset CRC and/or polyposis.

DV in *MSH2* and *MLH1* were responsible for the majority of cases of LS in our study, which is largely in line with previous retrospective studies in Sweden,^22^, ^23^, ^24^ and in studies from other Western countries.^25^ We found no DV in the *POLE* and *POLD1* exonuclease (proofreading) domains, but several VUS were detected in other regions of these genes. Possibly, as our understanding of the clinical impact expands, some of the current VUS located outside the proofreading domains may prove clinically relevant. Similarly, after variant re‐evaluation, no DV but several VUS remained in the genes associated with the hamartomatous polyposis syndromes.

More VUS than DV were initially identified in our cohort. However, after re‐evaluation approximately 45% of VUS were subsequently reclassified as likely benign variants. This shows that the ongoing sharing of data to open variant databases is crucial for improved variant classification. The importance of data sharing is further emphasized by the two cases of VUS in *PMS2* and *MLH1*, respectively, which were reassessed as causative for LS in our work. Although systematic reanalysis of sequence data has been reported to increase diagnostic yield,^26^, ^27^, ^28^, ^29^ clinical practice for reanalysis and reinterpretation remains to be established.^30^ Nevertheless, as outlined above we conclude that in the absence of multigene panel testing and follow‐up of VUS a total of five patients in our cohort could potentially have escaped the molecular genetic diagnosis of a Mendelian CRC syndrome.

During the study period, the routine laboratory diagnostic workup for simplex cases with an LS‐associated tumor diagnosed between ages 40–49 years included tumor tissue MMR protein immunohistochemical and mutation testing of *BRAF*, and subsequent targeted molecular genetic testing of any indicated MMR gene(s). Consequently, an unknown number of patients could have been diagnosed with LS through this alternative route, that is, outside the present cohort study. After variant re‐evaluation in an earlier retrospective study with emphasis on LS in Sweden, we observed only one such simplex case among a total of 52 patients with confirmed LS^24^ implying few additional cases diagnosed with LS during the period of the current study.

The role of heterozygous DV in *MUTYH* as risk alleles for CRC remains controversial.^20^, ^31^ We found eight heterozygote carriers for MAP, which corresponds to 0.94% of all patients in our cohort. The MAP carrier frequency in Northern Europe has been approximated to 1%–2%.^20^, ^31^ Since MAP carriers seem not to be accumulated in our cohort, our data do not support heterozygous DV in *MUTYH* as risk alleles for early‐onset CRC.

Introduction of paired sample tumor tissue DNA profiling for diagnostic and treatment decision purposes^32^, ^33^, ^34^ as well as exome or genome sequencing of noncancer patients now contribute to the identification of cancer predisposition alleles, either as sought or unsolicited findings. These rapidly emerging fields bypass the traditional triage for genetic testing of patients with suspected cancer predisposition based on age at diagnosis and family history. As technology develops and costs for testing drop it could be anticipated that a large proportion of germline cancer susceptibility soon will be identified through these novel diagnostic routes.

## 5 CONCLUSION

In summary, we describe the panorama and genetic variation of Mendelian CRC syndromes in a prospective cohort of patients with a suspected predisposition to CRC at the cancer genetics clinics in Sweden. Our data show that unified testing for the different Mendelian CRC syndromes as well as an intermittent reexamination of sequence data improve the diagnostic yield.

## CONFLICT OF INTEREST

The authors declare no conflict of interest.

## Supporting information


**APPENDIX S1** Supporting information

**APPENDIX S2** Supporting information

## Data Availability

The data that supports the findings of this study are available in the supplementary material of this article.
