# Supplementary material for: Merged testing for colorectal cancer syndromes and re‐evaluation of genetic variants improve diagnostic yield: Results from a nationwide prospective cohort
Source: Genes Chromosomes Cancer. 2022 May 2;61(10):585–91. doi: 10.1002/gcc.23049 (PMC9540764; doi:10.1002/gcc.23049)
Supplement: Supplementary file 1 — APPENDIX S1 Supporting information [file GCC-61-585-s002.docx]

**Supplementary Material 1.** Guidelines for routine clinical molecular genetic investigation for Mendelian predisposition to colorectal cancer

Clinical scenario A

Patient with clinical suspicion of herediatary polyposis syndrome (i.e., familial adenomatous polyposis (FAP) and *MUTYH*-associated polyposis (MAP), juvenile polyposis syndrome (JPS), *PTEN* hamartoma tumor syndrome (PHTS), Peutz-Jeghers syndrome (PJS), polymerase proofreading-associated polyposis (PPAP).

Gene panel: *APC*, *MUTYH*, *BMPR1A*, *SMAD4*, *PTEN*, *STK11*, *POLE*, *POLD1*

Clinical scenario B

Single patient, or patient within a cluster of first-degree relatives with

- 3 Lynch syndrome-related cancers*, at least one cancer diagnosed before age 60 years, or
- 2 Lynch syndrome-related cancer*, at least one cancer diagnosed before age 50 years, or
- 1 Lynch syndrome-related cancer* diagnosed before age 40 years.

Gene panel: *MLH1*, *MSH2*, *MSH6*, *PMS2*, *EPCAM*, *POLE*, *POLD1*

* Cancer of colon, rectum, uterus, ovary, stomach, small bowel, upper urinary tract
