# Supplementary material for: Merged testing for colorectal cancer syndromes and re‐evaluation of genetic variants improve diagnostic yield: Results from a nationwide prospective cohort
Source: Genes Chromosomes Cancer. 2022 May 2;61(10):585–91. doi: 10.1002/gcc.23049 (PMC9540764; doi:10.1002/gcc.23049)
Supplement: Supplementary file 2 — APPENDIX S2 Supporting information [file GCC-61-585-s001.docx]

**Supplementary Material 2.** Classification and reclassification of variants detected in cohort.

- Key for Class (variant classification as reported by the clinical laboratory at the time of analysis): 5; pathogenic, 4; likely pathogenic, 3; variant of unknown clinical significance.
- Key for Reclassification: - and +; weakened and strengthened likelihood for pathogenicity, respectively.
- Key for Reclassification criteria:

A; ClinVar (https://www.ncbi.nlm.nih.gov/clinvar/) classification as pathogenic or likely pathogenic variant (class 4-5) or benign or likely benign variant (class 1-2) according to expert panel

B; Presence together with pathogenic variant (class 4-5) (-)

C; Allele frequency in any population is >1/500 or >2 homozygous individuals if autosomal dominant inheritance in gnomAD (https://gnomad.broadinstitute.org/) (-)

D; Heterozygous carrier for autosomal recessive disease; Carrier of one variant in *MYTYH* (-)

E:I; Unstudied variation type; Variant i *POLE* or *POLD1* not situated in exonuclease domain & LoF variants (-)

E:II; Missense variant in *EPCAM* (-)

E:III; Intronic or silent variant not suspected to affect splice site (Alamut Visual; https://www.interactive-biosoftware.com/alamut-visual/) (-)

E:IV; variant located in untranslated region (-)

F; Consequence on protein. LoF where LoF is a known disease mechanism (+)

- Key for Result: LB; benign or likely benign variant (class 1-2), DV; pathogenic or likely pathogenic variant (class 4-5), VUS; variant of unknown clinical significance. Asterix denotes reclassified variant.

| **Patient** | **Gene** | **DNA sequence variant** | **Predicted protein** | **Type of DNA variant** | **Class** | **Reclassification** | **Reclassification criteria** | **Result** |
| --- | --- | --- | --- | --- | --- | --- | --- | --- |
| A004 | *APC* | c.721G>A | p.(Glu241Lys) | Missense | 3 |  |  | VUS |
| A008 | *MSH6* | c.1773A>G | p.(=) | Silent | 3 |  |  | VUS |
| A011 | *APC* | c.3927_3931del | p.(Glu1309Aspfs*4) | Frameshift | 5 |  |  | PV |
| A011 | *MUTYH* | c.821G>A | p.(Arg274Gln) | Missense | 3 | 3-- | B,C | LB* |
| A019 | *BMPR1A* | c.506_507insTCC | p.(Ile169_Phe170insPro) | Insertion | 3 |  |  | VUS |
| A022 | *MSH2* | c.646-1G>A | p.? | Splice site | 5 |  |  | PV |
| A024 | *APC* | c.3079T>A | p.(Tyr1027Asn) | Missense | 3 |  |  | VUS |
| A028 | *MSH2* | c.(?_-125)_(211+1_212-1)del | p.? | Deletion of exon(s) 1 | 5 |  |  | PV |
| A047 | *APC* | c.5612_5614dup | p.(Asp1871dup) | Duplication | 3 |  |  | VUS |
| A050 | *MSH2* | c.885C>G | p.(Asp295Glu) | Missense | 3 |  |  | VUS |
| A055 | *MUTYH* | c.536A>G | p.(Tyr179Cys) | Missense | 5 |  |  | PV |
| A056 | *MLH1* | c.-28A>G(;)-7C>T |  | 5' UTR | 3 | 3-- | C, E:IV | LB* |
| A060 | *MSH6* | c.2092C>T | p.(Gln698*) | Nonsense | 5 |  |  | PV |
| B003 | *APC* | c.5162G>A | p.(Gly1721Asp) | Missense | 3 |  |  | VUS |
| B004 | *APC* | c.4824_4827delinsTAC | p.(Lys1608Asnfs*42) | Frameshift | 5 |  |  | PV |
| B012 | *APC* | c.3386T>C | p.(Leu1129Ser) | Missense | 3 | 3- | C | LB* |
| B015 | *MUTYH* | c.312C>T | p.(=) | Silent | 3 | 3-- | D,E:III | LB* |
| B018 | *APC* | c.8068G>A | p.(Ala2690Thr) | Missense | 3 | 3- | C | LB* |
| B022 | *MSH6* | c.1346T>C | p.(Leu449Pro) | Missense | 5 |  |  | PV |
| B023 | *MLH1* | c.813del | p.(Leu272*) | Nonsense | 5 |  |  | PV |
| B024 | *MSH2* | c.2801C>T | p.(Thr943Met) | Missense | 3 |  |  | VUS |
| B026 | *APC* | c.2889T>C | p.(=) | Silent | 3 | 3- | E:III | LB* |
| B027 | *MSH6* | c.4001+12_4001+15del | p.? | Silent | 3 | 3- | E:III | LB* |
| B030 | *MSH2* | c.646-1G>A | p.? | Splice site | 5 |  |  | PV |
| B035 | *MLH1* | c.(1731+1_1732-1)_(1896+1_1897-1)del | p.? | Deletion of exon(s) 16 | 5 |  |  | PV |
| B037 | *APC* | c.481C>T | p.(Gln161*) | Nonsense | 5 |  |  | PV |
| B050 | *MLH1* | c.2059C>T | p.(Arg687Trp) | Missense | 5 |  |  | PV |
| B051 | *MSH2* | c.989T>C | p.(Leu330Pro) | Missense | 5 |  |  | PV |
| B056 | *MLH1* | c.2253dup | p.(Val752Serfs*3) | Duplication | 5 |  |  | PV |
| B061 | *MSH2* | c.2096C>A | p.(Ser699*) | Nonsense | 5 |  |  | PV |
| B068 | *MUTYH* | c.536A>G | p.(Tyr179Cys) | Missense | 5 |  |  | PV |
| B072 | *MSH6* | c.3543C>G | p.(Asp1181Glu) | Missense | 3 |  |  | VUS |
| B083 | *MSH2* | c.2038C>T | p.(Arg680*) | Nonsense | 5 |  |  | PV |
| B085 | *APC* | c.6779G>T | p.(Ser2260Ile) | Missense | 3 |  |  | VUS |
| B086 | *MUTYH* | c.536A>G(;)536A>G | p.(Tyr179Cys)(;)(Tyr179Cys) | Missense | 5,5 |  |  | PV, PV |
| B101 | *PMS2* | c.1437C>G | p.(His479Gln) | Missense | 3 | 3- | C | LB* |
| B104 | *MLH1* | c.2059C>T | p.(Arg687Trp) | Missense | 5 |  |  | PV |
| B105 | *PMS2* | c.300G>C | p.(Gln100His) | Missense | 3 |  |  | VUS |
| B106 | *MLH1* | c.2092_2093del | p.(Ser698Argfs*5) | Frameshift | 5 |  |  | PV |
| B109 | *MUTYH* | c.1214C>T | p.(Pro405Leu) | Missense | 5 |  |  | PV |
| B111 | *MSH2* | c.1147C>T | p.(Arg383*) | Nonsense | 5 |  |  | PV |
| B112 | *MUTYH* | c.1280_1289del | p.(Trp427Serfs*22) | Frameshift | 5 |  |  | PV |
| B113 | *MUTYH* | c.1187G>A | p.(Gly396Asp) | Missense | 5 |  |  | PV |
| B120 | *MSH2* | c.(1276+1_1277-1)_(1386+1_1387-1)del | p.? | Deletion of exon(s) 8 | 5 |  |  | PV |
| B122 | *MSH2* | c.1298T>A | p.(Leu433*) | Nonsense | 5 |  |  | PV |
| B128 | *MSH2* | c.1786_1788del | p.(Asn596del) | Deletion | 5 |  |  | PV |
| B131 | *MLH1* | c.-28A>G(;)-7C>T |  | 5' UTR | 3 | 3-- | C, E:IV | LB* |
| B131 | *POLD1* | c.1203C>A | p.(Phe401Leu) | Missense | 3 |  |  | VUS |
| B133 | *APC* | c.1902T>G | p.(Ser634Arg) | Missense | 4 |  |  | PV |
| B133 | *APC* | c.4472T>A | p.(Phe1491Tyr) | Missense | 3 | 3- | B | LB* |
| B136 | *APC* | c.2847G>T | p.(Met949Ile) | Missense | 3 |  |  | VUS |
| C001 | *MSH2* | c.1013G>T | p.(Gly338Val) | Missense | 4 |  |  | PV |
| C007 | *MSH6* | c.2818G>C | p.(Ala940Pro) | Missense | 3 |  |  | VUS |
| C016 | *POLD1* | c.2275G>A | p.(Val759Ile) | Missense | 3 | 3- | E:I | LB* |
| C017 | *POLE* | c.4523G>A | p.(Arg1508His) | Missense | 3 | 3- | E:I | LB* |
| C019 | *MUTYH* | c.1187G>A(;)c.1187G>A | p.(Gly396Asp)(;)p.(Gly396Asp) | Missense | 5,5 |  |  | PV, PV |
| C021 | *APC* | c.5140G>A | p.(Asp1714Asn) | Missense | 3 |  |  | VUS |
| C025 | *MUTYH* | c.536A>G(;)c.734G>A | p.(Tyr179Cys)(;)(Arg245His) | Missense | 5,5 |  |  | PV, PV |
| C030 | *POLD1* | c.328C>T | p.(Pro110Ser) | Missense | 3 | 3- | E:I | LB* |
| C031 | *MSH2* | c.1982_1985del | p.(Lys661Argfs*23) | Frameshift | 5 |  |  | PV |
| C034 | *EPCAM* | c.(858+1_859-1)_(945+?)del | p.? | Deletion of exon(s) 8-9 | 5 |  |  | PV |
| C034 | *BMPR1A* | c.1330T>C | p.(Cys444Arg) | Missense | 4 |  | B | VUS |
| C037 | *MUTYH* | c.1187G>A | p.(Gly396Asp) | Missense | 5 |  |  | PV |
| C044 | *MSH6* | c.3647-2A>C | p.? | Splice site | 5 |  |  | PV |
| C045 | *APC* | c.8389A>G | p.(Ser2797Gly) | Missense | 3 |  |  | VUS |
| C046 | *PMS2* | c.2113G>A | p.(Glu705Lys) | Missense | 5 |  |  | PV |
| C053 | *APC* | c.6724A>G | p.(Ser224Gly) | Missense | 3 |  |  | VUS |
| C059 | *POLE* | c.861T>A | p.(Asp278Glu) | Missense | 3 | 3- | C | LB* |
| C066 | *MLH1* | c.203T>A | p.(Ile68Asn) | Missense | 4 |  |  | PV |
| C068 | *PMS2* | c.137G>T(;)2113G>A | p.(Ser46Ile)(;)(Glu705Lys) | Missense | 4,5 |  |  | PV, PV |
| C072 | *APC* | c.1571G>A | p.(Gly524Asp) | Missense | 3 |  |  | VUS |
| C074 | *MLH1* | c.492A>C | p.(Lys164Asn) | Missense | 3 |  |  | VUS |
| C075 | *PMS2* | c.137G>T | p.(Ser46Ile) | Missense | 4 |  |  | PV |
| C076 | *APC* | c.2555T>A | p.(Leu852*) | Nonsense | 5 |  |  | PV |
| C078 | *MLH1* | c.304G>A | p.(Glu102Lys) | Missense | 3 | 4 | A | PV* |
| C078 | *MLH1* | c.595G>C | p.(Glu199Gln) | Missense | 3 | 3- | B | LB* |
| C078 | *APC* | c.295C>T | p.(Arg99Trp) | Missense | 3 | 3- | B | LB* |
| C082 | *MSH6* | c.390T>G | p.(His130Gln) | Missense | 3 |  |  | VUS |
| C083 | *POLE* | c.2090C>G | p.(Pro697Arg) | Missense | 3 | 3- | E:I | LB* |
| C089 | *MLH1* | c.546-2A>G | p.? | Splice site | 5 |  |  | PV |
| C093 | *PMS2* | c.379G>A | p.(Ala127Thr) | Missense | 3 | 3- | C | LB* |
| C098 | *MSH6* | c.3668A>T | p.(Asp1223Val) | Missense | 3 |  |  | VUS |
| C100 | *POLE* | c.4523G>A | p.(Arg1508His) | Missense | 3 | 3- | E:I | LB* |
| C101 | *APC* | c.3920T>A | p.(Ile1307Lys) | Missense | 3 | 3- | C | LB* |
| C107 | *MSH6* | c.2779dup | p.(Ile927Asnfs*8) | Frameshift | 5 |  |  | PV |
| C110 | *MLH1* | c.1964T>C | p.(Ile655Thr) | Missense | 3 |  |  | VUS |
| C112 | *POLE* | c.5542C>T | p.(Leu1848Phe) | Missense | 3 | 3- | E:I | LB* |
| C116 | *MLH1* | c.2059C>T | p.(Arg687Trp) | Missense | 5 |  |  | PV |
| C119 | *MSH2* | c.289C>T | p.(Gln97*) | Nonsense | 5 |  |  | PV |
| C121 | *EPCAM* | c.831A>G | p.(Ile277Met) | Missense | 3 | 3- | E:II | LB* |
| C121 | *GALNT12* | c.115G>A | p.(Ala39Thr) | Missense | 3 |  |  | VUS |
| C123 | *MSH6* | c.390T>G | p.(His130Gln) | Missense | 3 |  |  | VUS |
| C124 | *POLE* | c.2090C>G | p.(Pro697Arg) | Missense | 3 | 3- | E:I | LB* |
| C134 | *MSH6* | c.362G>A | p.(Arg121His) | Missense | 3 |  |  | VUS |
| C136 | *BMPR1A* | c.1439G>A | p.(Arg480Gln) | Missense | 3 |  |  | VUS |
| C139 | *SMAD4* | c.1573A>G | p.(Ile525Val) | Missense | 3 | 3- | C | LB* |
| C142 | *POLD1* | c.1128C>A | p.(Asp376Glu) | Missense | 3 |  |  | VUS |
| C143 | *APC* | c.3358G>T | p.(Gly1120*) | Nonsense | 5 |  |  | PV |
| C143 | *POLE* | c.4411C>T | p.(Arg1471Cys) | Missense | 3 | 3- | E:I | LB* |
| C150 | *POLE* | c.139C>T | p.(Arg47Trp) | Missense | 3 | 3- | E:I | LB* |
| C156 | *MUTYH* | c.337T>C | p.(Trp113Arg) | Missense | 3 | 3- | D | LB* |
| C157 | *MLH1* | c.67G>T | p.(Glu23*) | Nonsense | 5 |  |  | PV |
| C157 | *POLD1* | c.2933G>A | p.(Arg978His) | Missense | 3 | 3- | E:I | LB* |
| C160 | *MSH2* | c.1088_1093del | p.(Val363_Glu364del) | Deletion | 3 |  |  | VUS |
| C167 | *MSH6* | c.3606dup | p.(His1203Alafs*12) | Frameshift | 5 |  |  | PV |
| C169 | *MSH6* | c.1190_1191del | p.(Tyr397Cysfs*3) | Frameshift | 5 |  |  | PV |
| C170 | *MSH6* | c.3394G>C | p.(Val1132Leu) | Missense | 3 |  |  | VUS |
| C173 | *MUTYH* | c.916C>T | p.(Arg306Cys) | Missense | 3 | 3- | D | LB* |
| C174 | *PMS2* | c.154A>G | p.(Thr52Ala) | Missense | 3 |  |  | VUS |
| C181 | *EPCAM* | c.267G>C | p.(Gln89His) | Missense | 3 | 3- | E:II | LB* |
| C194 | *APC* | c.8389A>G | p.(Ser2797Gly) | Missense | 3 |  |  | VUS |
| C195 | *POLE* | c.5492T>C | p.(Leu1831Pro) | Missense | 3 | 3- | E:I | LB* |
| C198 | *PMS2* | c.(23+1_24-1)_(988+1_989-1)del | p.? | Deletion of exon(s) 2-9 | 5 |  |  | PV |
| C199 | *MLH1* | c.350C>T | p.(Thr117Met) | Missense | 5 |  |  | PV |
| C208 | *PMS2* | c.711A>G | p.(=) | Silent | 3 | 3- | E:III | LB* |
| C220 | *MSH6* | c.2713T>A | p.(Leu905Met) | Missense | 3 |  |  | VUS |
| C222 | *APC* | c.3014C>T | p.(Ala1005Val) | Missense | 3 |  |  | VUS |
| C223 | *MSH2* | c.2006-1G>T | p.? | Splice site | 5 |  |  | PV |
| C226 | *POLD1* | c.2429C>T | p.(Ala810Val) | Missense | 3 | 3- | E:I | LB* |
| C228 | *MSH6* | c.2183A>C | p.(Lys728Thr) | Missense | 3 |  |  | VUS |
| C229 | *POLE* | c.4055G>C | p.(Gly1352Ala) | Missense | 3 | 3- | E:I | LB* |
| C232 | *MSH2* | c.1587del | p.(Glu530Lysfs*13) | Frameshift | 5 |  |  | PV |
| C234 | *POLE* | c.1846C>T | p.(Arg616Cys) | Missense | 3 | 3- | E:I | LB* |
| D002 | *MLH1* | c.91_92delinsTG | p.(Ala31Cys) | Missense | 3 |  |  | VUS |
| D003 | *MLH1* | c.1360G>C | p.(Gly454Arg) | Missense | 3 |  |  | VUS |
| D004 | *MSH2* | c.2634+5G>A | p.? | Splice site | 4 |  |  | PV |
| D009 | *MSH2* | c.1226_1227del | p.(Gln409Argfs*7) | Frameshift | 5 |  |  | PV |
| D018 | *MSH6* | c.2667G>T | p.(Gln889His) | Missense | 3 | 3- | C | LB* |
| D021 | *MSH2* | c.2379G>T | p.(Gln793His) | Missense | 3 |  |  | VUS |
| D021 | *APC* | c.2871G>A | p.(=) | Silent | 3 | 3- | E:III | LB* |
| D023 | *MSH6* | c.1474A>G | p.(Met492Val) | Missense | 3 |  |  | VUS |
| D025 | *MSH6* | c.3848_3850dup | p.(Ile1283dup) | Duplication | 3 |  |  | VUS |
| D036 | *MSH6* | c.10C>G | p.(Gln4Glu) | Missense | 3 |  |  | VUS |
| D042 | *MLH1* | c.2059C>T | p.(Arg687Trp) | Missense | 5 |  |  | PV |
| D044 | *MSH6* | c.4001+2T>C | p.? | Splice site | 5 |  |  | PV |
| D056 | *POLD1* | c.1573C>T | p.(Arg525Trp) | Missense | 3 | 3- | E:I | LB* |
| D060 | *APC* | c.295C>T | p.(Arg99Trp) | Missense | 3 |  |  | VUS |
| D068 | *GALNT12* | c.1638A>G | p.(=) | Silent | 3 |  |  | VUS |
| D070 | *MLH1* | c.546-2A>G | p.? | Splice site | 5 |  |  | PV |
| D070 | *STK11* | c.1130C>T | p.(Ala377Val) | Missense | 3 | 3- | B | LB* |
| D073 | *MLH1* | c.2059C>T | p.(Arg687Trp) | Missense | 5 |  |  | PV |
| D082 | *MLH1* | c.2059C>T | p.(Arg687Trp) | Missense | 5 |  |  | PV |
| D085 | *MSH6* | c.2400del | p.(Val801Cysfs*9) | Frameshift | 5 |  |  | PV |
| D088 | *MUTYH* | c.536A>G | p.(Tyr179Cys) | Missense | 5 |  |  | PV |
| D092 | *MSH6* | c.377C>G | p.(Ser126*) | Nonsense | 5 |  |  | PV |
| D094 | *PMS2* | c.58C>G | p.(Arg20Gly) | Missense | 3 |  |  | VUS |
| D099 | *MLH1* | c.546-2A>G | p.? | Splice site | 5 |  |  | PV |
| D100 | *MSH2* | c.80C>G | p.(Pro27Arg) | Missense | 3 | 3- | B | LB* |
| D100 | *MSH2* | c.1986_1987del | p.(Gln662Hisfs*13) | Frameshift | 5 |  |  | PV |
| D103 | *MSH6* | c.3656C>T | p.(Thr1219Ile) | Missense | 4 |  |  | PV |
| E001 | *MSH2* | c.2459-2A>T | p.? | Splice site | 4 |  |  | PV |
| E004 | *MSH2* | c.943-2A>G | p.? | Splice site | 4 |  |  | PV |
| E006 | *MSH2* | c.2399del | p.(Leu800Hisfs*12) | Frameshift | 4 |  |  | PV |
| E007 | *PMS2* | c.52A>G | p.(Ile18Val) | Missense | 3 | 2 | A | LB* |
| E013 | *MSH6* | c.3261del | p.(Phe1088Serfs*2) | Frameshift | 5 |  |  | PV |
| E014 | *MUTYH* | c.1214C>T(;)1214C>T | p.(Pro405Leu)(;)(Pro405Leu) | Missense | 5,5 |  |  | PV, PV |
| E017 | *MSH6* | c.1063G>A | p.(Gly355Ser) | Missense | 3 | 3- | C | LB* |
| E018 | *PMS2* | c.2113G>A | p.(Glu705Lys) | Missense | 4 |  |  | PV |
| E020 | *PMS2* | c.857A>G | p.(Asp286Gly) | Missense | 3 |  |  | VUS |
| E020 | *BMPR1A* | c.275G>A | p.(Gly92Glu) | Missense | 3 |  |  | VUS |
| E021 | *MSH2* | c.(211+1_212-1)_(645+1_646-1)del | p.? | Deletion of exon(s) 2-3 | 5 |  |  | PV |
| E023 | *MSH2* | c.1982_1985del | p.(Lys661Argfs*23) | Frameshift | 5 |  |  | PV |
| E030 | *MSH2* | c.(?_-125)_(1386+1_1387-1)del | p.? | Deletion of exon(s) 1-8 | 5 |  |  | PV |
| E034 | *APC* | c.147_150del | p.(Lys49Asnfs*20) | Frameshift | 5 |  |  | PV |
| E035 | *MSH6* | c.59C>T | p.(Ala20Val) | Missense | 3 | 2 | A | LB* |
| E038 | *POLE* | c.691C>T | p.(Arg231Cys) | Missense | 3 |  |  | VUS |
| E039 | *MSH6* | c.3802-3_3825dup | p.(Cys1275_Glu1276ins9) | Duplication | 4 |  |  | PV |
| E045 | *MSH2* | c.759G>A | p.(Met253Ile) | Missense | 3 |  |  | VUS |
| E046 | *APC* | c.3848_3850dup | p.(Ala1283dup) | Duplication | 3 |  |  | VUS |
| E046 | *APC* | c.5627G>T | p.(Arg1876Met) | Missense | 3 |  |  | VUS |
| E047 | *APC* | c.1775T>G | p.(Leu592*) | Nonsense | 5 |  |  | PV |
| E049 | *MSH2* | c.(1076+1_1077-1)_(1386+1_1387-1)del | p.? | Deletion of exon(s) 7-8 | 5 |  |  | PV |
| E051 | *APC* | c.721G>A | p.(Glu241Lys) | Missense | 3 |  |  | VUS |
| E052 | *BMPR1A* | c.590A>C | p.(Glu197Ala) | Missense | 3 |  |  | VUS |
| E053 | *BMPR1A* | c.712C>G | p.(Arg238Gly) | Missense | 3 |  |  | VUS |
| E055 | *APC* | c.7379C>T | p.(Ala2460Val) | Missense | 3 |  |  | VUS |
| E064 | *MLH1* | c.[(-7C>T)(;)(-28A>G)] | p.(=) | 5' UTR | 3 | 3-- | C, E:IV | LB* |
| E066 | *PMS2* | c.686_687del | p.(Ser229Cysfs*19) | Frameshift | 5 |  |  | PV |
| E070 | *MLH1* | c.376T>A | p.(Tyr126Asn) | Missense | 3 |  |  | VUS |
| E084 | *MUTYH* | c.536A>G(;)c.933+3A>C | p.[(Tyr179Cys)(;)(Gly264Trpfs*7)] | Missense, Splice site | 5,5 |  |  | PV, PV |
| E085 | *PMS2* | c.(23+1_24-1)_(988+1_989-1)del | p.? | Deletion of exon(s) 2-9 | 5 |  |  | PV |
| E087 | *PMS2* | c.2350G>A | p.(Asp784Asn) | Missense | 3 | 3- | C | LB* |
| E088 | *MUTYH* | c.734G>A(;)1437_1439del | p.[(Arg245His)(;)(Glu480del)] | Missense, Deletion | 5,5 |  |  | PV, PV |
| E089 | *MSH2* | c.1490_1492del | p.(Ile497del) | Deletion | 4 |  |  | PV |
| E095 | *MSH6* | c.1691C>G | p.(Ser564*) | Nonsense | 5 |  |  | PV |
| E102 | *MSH2* | c.(1076+1_1077-1)_(1386+1_1387-1)del | p.? | Deletion of exon(s) 7-8 | 5 |  |  | PV |
| E104 | *MLH1* | c.244A>G | p.(Thr82Ala) | Missense | 4 |  |  | PV |
| E107 | *APC* | c.295C>T | p.(Arg99Trp) | Missense | 3 |  |  | VUS |
| E110 | *SMAD4* | c.608C>T | p.(Pro203Leu) | Missense | 3 |  |  | VUS |
| E113 | *APC* | c.4072G>A | p.(Ala1358Thr) | Missense | 3 |  |  | VUS |
| E118 | *POLE* | c.861T>A | p.(Asp287Glu) | Missense | 3 | 3- | C | LB* |
| E133 | *APC* | c.531+1G>A | p.? | Splice site | 5 |  |  | PV |
| E135 | *PMS2* | c.2113G>A | p.(Glu705Lys) | Missense | 4 |  |  | PV |
| E138 | *MUTYH* | c.1187G>A(;)1214C>T | p.[(Gly396Asp)(;)(Pro405Leu)] | Missense | 5,5 |  |  | PV, PV |
| E141 | *PMS2* | c.2149G>A | p.(Val717Met) | Missense | 3 | 3- | B | LB* |
| E141 | *PMS2* | c.2192_2196del | p.(Leu731Cysfs*3) | Frameshift | 5 |  |  | PV |
| E143 | *MSH6* | c.3146C>T | p.(Ser1049Phe) | Missense | 3 |  |  | VUS |
| E149 | *APC* | c.221-2A>G | p.? | Splice site | 5 |  |  | PV |
| E152 | *APC* | c.2868C>A | p.(Tyr956*) | Nonsense | 5 |  |  | PV |
| E158 | *MUTYH* | c.934-2A>G | p.? | Splice site | 3 | 3- | D | LB* |
| E162 | *MSH2* | c.2320A>G | p.(Ile774Val) | Missense | 3 |  |  | VUS |
| E172 | *MUTYH* | c.352G>T | p.(Glu118*) | Nonsense | 5 |  |  | PV |
| E177 | *APC* | c.8060C>T | p.(Ser2687Leu) | Missense | 3 |  |  | VUS |
| F004 | *MUTYH* | c.536G>A(;)536G>A | p.(Tyr179Cys) | Missense | 5,5 |  |  | PV, PV |
| F004 | *APC* | c.8068G>A | p.(Ala2690Thr) | Missense | 3 | 3--- | A, B, C | LB* |
| F005 | *PMS2* | c.1437C>G | p.(His479Gln) | Missense | 3 | 3- | C | LB* |
| F008 | *APC* | c.8383G>A | p.(Ala2795Thr) | Missense | 3 |  |  | VUS |
| F009 | *MUTYH* | c.493G>A | p.(Ala165Thr) | Missense | 3 | 3- | D | LB* |
| F013 | *MLH1* | c.546-2A>G | p.? | Splice site | 5 |  |  | PV |
| F016 | *MSH2* | c.138C>G | p.(His46Gln) | Missense | 3 | 2 | A | LB* |
| F021 | *MLH1* | c.1379A>C | p.(Glu460Ala) | Missense | 3 |  |  | VUS |
| F025 | *MSH6* | c.3103C>T | p.(Arg1035*) | Nonsense | 5 |  |  | PV |
| F027 | *MSH2* | c.703A>G | p.(Lys235Glu) | Missense | 3 |  |  | VUS |
| F029 | *MSH6* | c.2239C>T | p.(=) | Silent | 3 | 3- | E:III | LB* |
| F035 | *APC* | c.295C>T | p.(Arg99Trp) | Missense | 3 |  |  | VUS |
| F049 | *MSH6* | c.1754T>C | p.(Leu585Pro) | Missense | 3 |  |  | VUS |
| F050 | *APC* | c.7478T>C | p.(Leu249Pro) | Missense | 3 |  |  | VUS |
| F051 | *PMS2* | c.2382dup | p.(Gly795Trpfs*29) | Frameshift | 3 | 3+ | F | PV* |
| F052 | *STK11* | c.1180G>A | p.(Gly394Ser) | Missense | 3 |  |  | VUS |
| F058 | *APC* | c.3511C>T | p.(Arg1171Cys) | Missense | 3 | 3- | C | LB* |
| F062 | *SMAD4* | c.1573A>G | p.(Ile525Val) | Missense | 3 | 3- | C | LB* |
| F073 | *MSH6* | c.900dup | p.(Lys301Glufs*11) | Frameshift | 5 |  |  | PV |
| F095 | *POLE* | c.20_29del | p.(Glys7Alafs*44) | Frameshift | 3 | 3- | E:I | LB* |
| F100 | *APC* | c.3927_3931del | p.(Glu1309Aspfs*4) | Frameshift | 5 |  |  | PV |
| F100 | *PMS2* | c.1437C>G | p.(His479Gln) | Missense | 3 | 3-- | A, C | LB* |
| F100 | *MSH2* | c.2718A>G | p.(Ile906Met) | Missense | 3 | 3-- | A, C | LB* |
| F104 | *MLH1* | c.2141G>A | p.(Trp714*) | Nonsense | 5 |  |  | PV |
| F108 | *PMS2* | c.(23+1_24-1)_(988+1_989-1)del | p.? | Deletion of exon(s) 2-9 | 5 |  |  | PV |
| F110 | *MSH2* | c.2680dup | p.(Met894Asnfs*5) | Frameshift | 5 |  |  | PV |
| F120 | *APC* | c.5894A>C | p.(His1965Pro) | Missense | 3 |  |  | VUS |
| F128 | *MLH1* | c.199G>A | p.(Gly67Arg) | Missense | 5 |  |  | PV |
| F129 | *POLE* | c.89C>A | p.(Ser30*) | Nonsense | 3 | 3- | E:I | LB* |
